# Evalution of anti-ulcer activity of *Polyalthia longifolia* (Sonn.) Thwaites in experimental animals

**DOI:** 10.4103/0253-7613.42306

**Published:** 2008-06

**Authors:** P. Malairajan, Geetha Gopalakrishnan, S. Narasimhan, K. Jessi Kala Veni

**Affiliations:** Rofel Shri GM Bilakhia College of Pharmacy, Vapi (West)-396 191, Gujarat State, India; 1 R and D Leader, Schering Plough Link, Singapore Branch, Tuas West drive, Singapore 638 403; 2 Asthagiri Herbal Research foundation, Tambaram Sanatorium, Chennai-600 053, Tamil Nadu, India

**Keywords:** Aspirin pylorous ligation ulceration, HCl-Ethanol ulcer, *Polyalthia longifolia*, stress ulcer

## Abstract

**Objective:**

To evaluate the anti-ulcer activity of ethanol extract of leaves of *Polyalthia longifolia* (Sonn.) Thwaites.

**Materials and Methods:**

The ethanol extract of *Polyalthia longifolia* was investigated for its anti-ulcer activity against aspirin plus pylorous ligation induced gastric ulcer in rats, HCl -Ethanol induced ulcer in mice and water immersion stress induced ulcer in rats at 300 mg/kg body weight.p.o.

**Results:**

A significant (*P* < 0.01, *P* < 0.001) anti ulcer activity was observed in all the models. Pylorous ligation showed significant (*P*< 0.01) reduction in gastric volume, free acidity and ulcer index as compared to control. It also showed 89.71% ulcer inhibition in HCl- Ethanol induced ulcer and 95.3% ulcer protection index in stress induced ulcer.

**Conclusion:**

This present study indicates that *P. longifolia* leaves extract have potential anti ulcer activity in the three models tested.

In traditional medicines various herbal preparations are being used for treating duodenal ulcers.[1] *Polyalthia longifolia* (Sonn.) Thwaites (PL) (Family: Annonaceae) is a tall handsome evergreen tree and it is cultivated all over India. The plant has been used in traditional system of medicine for the treatment of fever, skin diseases, diabetes, hypertension and helminthiasis.[2] A number of biologically active compounds have been isolated from this plant.[3–6] The plant extract and isolated compounds were studied for various biological activities like antibacterial activity, cytotoxicity, antifungal activity.[7–11] The present study was undertaken to evaluate the anti ulcer activity of ethanol extract of *P. longifolia* (PL) against aspirin plus pylorous ligation induced gastric ulcer, HCl - Ethanol induced ulcer and water immersion stress induced ulcer in rats.

## Materials and Methods

### Plant material

The leaves of *Polyalthia longifolia* was collected from Kancheepuram Dist, South India during September 2000 and authenticated by Professor P. Jeyaraman, Plant Anatomy Research Centre, Tambaram, Chennai-45. The specimen voucher was deposited in the Asthagiri Herbal Research Foundation [AHRF 09] Chennai-59.

The leaves of *P. longifolia* were air-dried and powdered plant material was extracted by maceration with ethanol for 72h. The extract was concentrated using rotary vacuum to get the solid mass. The yield obtained was 13.73%. The extract, ranitidine, sucralfate and omeprazole were suspended in 1% sodium carboxy methyl cellulose (SCMC) and used for anti-ulcer studies.

### Animals

*Wistar* albino rats of both sex and Swiss albino mice were obtained from Tamil Nadu Veterinary College and Research Institute, Chennai. The animals were housed in polypropylene cages at 24±2°C and fed with commercial pellet diet and water *ad libitum.* All animal experiments were carried out in accordance with the guidelines of CPCSEA and the study was approved by the Institutional Animal Ethics Committee.

### Acute oral toxicity studies

A safe oral dose of the extract was determined by acute oral toxic class method of Organization of Economic Co-Operation and Development (OECD) as per 423 guidelines.[12] The plant reported to contain the antiulcerogenic principle liriodenine and annonains. So the present study followed three approaches of antiulcerogenic mechanism of the plant extract:

1. Aspirin plus pylorus ligation induced gastric ulcer in rats (antisecretory mechanism).
2. HCl - Ethanol induced ulcer (cytoprotective mechanism).
3. Water immersion stress induced ulcer in rats (proton pump inhibition mechanism).

For each model the corresponding standard drug such as ranitidine, sucralfate and omeprazole was used.

### Aspirin plus pylorus ligation induced gastric ulcer in rats

The Wistar albino rats weighing 100-200 g of either sex were divided into 3 groups, each group consists of 6 animals. All the animals received 200 mg/kg of aspirin once daily for three days. Group 1 served as control received 1.0 ml/kg p.o 1% SCMC, group 2 treated with 50 mg/kg, p.o ranitidine as standard, group 3 treated with 300 mg/kg, p.o. ethanol extract of PL. On the fourth day pylorus part was ligated following 36h fasting.[13] Four hours after the pyloric ligation the animals were sacrificed by decapitation. The stomach was opened and the ulcer index was determined.[14] The gastric content was titrated against 0.01 N NaOH to find out the free acidity and total acidity.[15]

### Ulcer lesion Index method: HCl - Ethanol induced ulcer

Swiss albino mice weighing 24-30 g of either sex were divided into 3 groups, each group consists of 6 animals. Group 1 served as a control received 1.0 ml/kg p.o 1% SCMC, group 2 received 100 mg/kg, p.o sucralfate as standard control; group 3 received 300 mg/kg, p.o ethanol extract of PL. After 1h all the animals were treated with 0.2 ml of HCl - Ethanol mixture p.o (0.3 M Hydrochloric acid and ethanol 60%) to induce gastric ulcer. Animals were sacrificed by cervical dislocation one hour after administration of HCl - Ethanol mixture. The stomach was excised and lesion index was determined by measuring each lesion in mm along its greater length.[16]

### Water immersion stress induced ulcer in rats

Stress ulcers were induced by forced swimming in the glass cylinder[17] (height 45 cm, diameter 25 cm) containing water to the height of 35 cm maintained at 25°C for 3h. Animals were fasted for 24h prior to the experiment and divided in to 3 groups with 6 animals in each group. Group 1 received 1.0 ml/kg p.o 1% SCMC as vehicle control, group 2 treated with 20 mg/kg, p.o. omeprazole as standard control, group 3 received 300 mg/kg, p.o ethanol extract of PL. After the drug treatment animals were allowed to swim in water for 3 h. The stomach of each animal was removed and the extent of gastric damage was assessed as described by Alphine and Word.[18] 

### Statistical analysis

The statistical analysis of all the results was carried out using one-way ANOVA followed by Dunnets multiple comparisons using graph pad in stat 3 and all the results obtained in the study were compared with the vehicle control group.

## Results

In aspirin plus pylorous ligation induced gastric ulcer the ethanol extract of PL showed significant (*P*< 0.01) reduction in gastric volume, free acidity and ulcer score (% ulcer inhibition 53.69) as compared to control [Table 1]. From Table 2 it can be observed that the number of lesions in HCI-Ethanol induced peptic ulcer group was significantly high and the ethanol extract of PL pretreated group depicted marked reduction (*P* < 0.01) in gastric lesion (% ulcer inhibition 89.71) as compared to control [Table 2].

**Table 1:** Effect of ethanol extract of *P. longifolia* on gastric secretion, acidity, pH and ulcer score in aspirin plus pylorus ligated rats

*Treatment (mg/kg b.wt)*	*Volume of gastric secretion (ml/100 g)*	*Free acidity (mEq/l/100 g)*	*Total acidity (mEq/l/100 g)*	*pH*	*Ulcer score*	*ulcer inhibition (%)*
Vehicle control (1%SCMC)	2.633±0.042	225.00±6.124	555.00±7.500	2.200±0.163	3.600±0.200	-
Ranitidine 50	1.317±0.172	148.75±13.475**	492.50±20.736*	3.167±0.166*	0.166±0.166**	95.37
*P. longifolia* 300	0.983±0.083**	135.00±10.782**	552.50±10.724	2.333±0.210	1.667.±0.307**	53.69

Values are expressed as mean ± SEM (n=6).

* *P*<0.05

** *P*<0.01 as compared to control

**Table 2:** Effect of ethanol extract of *P. longifolia* against HCl – Ethanol induced gastric lesion in mice (n=6)

*Treatment*	*Dose (mg/kg b.wt)*	*Gastric lesion (Mean ± SEM)*	*Ulcer inhibition (%)*
Control	1% SCMC	22.667±3.509	-
Sucralfate	100	1.167±0.5426**	94.85
*P. longifolia*	300	2.333±.557**	89.71

** *P* <0.05 as compared to control

In water immersion stress induced ulcer the mean score value of ulcer inhibition was found to be significant (*P* < 0.001) and the % ulcer inhibition was 95.3 [Table 3].

**Table 3:** Effect of ethanol extract of *P. longifolia* on water immersion stress induced ulcer score value expressed in mean ± SEM in rats (n=6)

*Treatment*	*Dose (mg/kg b.wt)*	*Mean ulcer score*	*Ulcer inhibition (%)*
Vehicle control	1% SCMC	143.3 ± 12.01	-
Omeprazole	20	0.0 ± 0.0***	100
*P. longifolia*	300	6.66 ± 2.10***	95.3

*** *P*<0.001 as compared to control

## Discussion

In aspirin plus pylorus ligation induced gastric ulcer model the ethanol extracts of *P. longifolia* reduced the gastric volume, free acidity, total acidity and ulcer index thus showing the anti-secretory mechanism involved in the extracts for their anti-ulcerogenic activity. Ulcer index parameter was used for the evaluation of anti-ulcer activity since ulcer formation is directly related to factors such as gastric volume, free and total acidity. In case of vehicle control, aspirin plus pylorus ligation increased the acid secretion, which in turn caused increase in gastric volume, low pH, increased free and total acidity resulting into increase in ulcer index.[19]

HCl-Ethanol induced gastric damage is possibly through leukotrienes production and 5-lipooxygenase pathway. Prostaglandins also play a role in ethanol-induced ulcer. It has been shown that drugs which are effective against HCI-Ethanol induced gastric lesions can possess gastric mucosal membrane protective action. The protective effect of the extract *P. longifolia* against the gastric damage may be due to their action against 5-lipooxygenase pathway. The cytoprotective action probably stimulates the prostaglandin synthesis, which in turn protects the gastric mucosa.

Water immersion stress is one of the best models of stress in rats to induce ulcer. The model provides both emotional stress as well as physiological stress to the animal. In case of water immersion induced stress in rats, the extract showed significant (*P*< 0.001) ulcer inhibition. Preliminary phytochemical investigations showed the presence of alkaloids and terpenoids, hence the antiulcer activity of PL in this experimental model may be due to the above mentioned chemicals. The results indicate that *P. longifolia* extract produced antiulcerogenic effects possessing antisecretory, cytoprotective and proton pump inhibition mechanism. This study indicates that *P. longifolia* extract has a potertial anti ulcer activity. However, further study is required to isolate the active molecule responsible for the activity.
